# The structures of ordered defects in thiocyanate analogues of Prussian Blue†

**DOI:** 10.1039/d0sc01246g

**Published:** 2020-04-09

**Authors:** Matthew J. Cliffe, Evan N. Keyzer, Andrew D. Bond, Maxwell A. Astle, Clare P. Grey

**Affiliations:** Department of Chemistry, University of Cambridge Lensfield Road Cambridge CB2 1EW UK; School of Chemistry, University of Nottingham University Park Nottingham NG7 2RD UK matthew.cliffe@nottingham.ac.uk

## Abstract

We report the structures of six new divalent transition metal hexathiocyanatobismuthate frameworks with the generic formula 
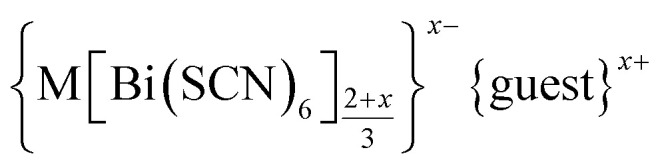
, M = Mn, Co, Ni and Zn. These frameworks are defective analogues of the perovskite-derived trivalent transition metal hexathiocyanatobismuthates M^III^[Bi(SCN)_6_]. The defects in these new thiocyanate frameworks order and produce complex superstructures due to the low symmetry of the parent structure, in contrast to the related and more well-studied cyanide Prussian Blue analogues. Despite the close similarities in the chemistries of these four transition metal cations, we find that each framework contains a different mechanism for accommodating the lowered transition metal charge, making use of some combination of Bi(SCN)_6_^3−^ vacancies, M_Bi_ antisite defects, water substitution for thiocyanate, adventitious extra-framework cations and reduced metal coordination number. These materials provide an unusually clear view of defects in molecular framework materials and their variety suggests that similar richness may be waiting to be uncovered in other hybrid perovskite frameworks.

## Abstract 2

We report the structures of six new divalent transition metal hexathiocyanatobismuthate Prussian Blue analogues frameworks, which contain complex ordered defect structures.

## Graphical abstract

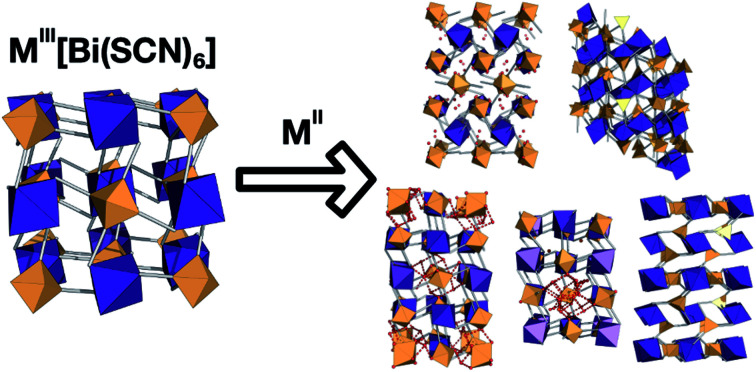

## 1 Introduction

Defects are ubiquitous in functional materials and play a critical role in materials as simple as the binary rocksalt oxides^1,2^ and as complex as high-temperature superconductors.^3^ The importance of vacancy chemistry to molecular framework perovskites is becoming increasingly clear^4^ in many important families, including the hybrid metal-halide semiconductors,^5,6^ cyanide Prussian Blue analogue battery cathodes^7,8^ and magnetic formate perovskites,^9,10^ as the number of studies making use of defect-engineering in these materials grows. Perhaps the most widespread strategy for introducing defects is aliovalent doping, where an ion is replaced by an ion with a different charge. Thus far, cationic dopants have been the most widely used and have been introduced onto both the A, *e.g.* NH_3_CH_2_CH_2_NH_3_^2+^ (EN^2+^) substitutes for CH_3_NH_3_^+^ (MA^+^) in CH_3_NH_3_[PbI_3_] to produce ‘hollow’ perovskites,^5^ and the B site, *e.g.* Fe^2+^ substitutes for Fe^3+^ in Prussian Blue.^11^ In hybrid perovskites, these charged point defects are typically compensated by low-energy point defects of the opposite charge, most commonly vacancies, rather than by electronic defects.^12^ For example, in the ‘hollow’ lead iodide perovskites, the additional A site charge is compensated primarily by vacancies on the B site, and can be represented in the Kröger–Vink defect notation by1
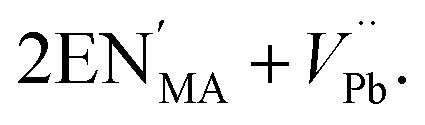


However, this simplified defect reaction only provides a partial description of the structure of these defective materials. Bulk chemical analysis indicates that in addition to B site vacancies ‘hollow’ perovskites contain a substantial concentration of iodide vacancies, as much as 6%, and diffraction measurements indicate the presence of local defect-ordering beyond that visible in the average structure.^5^ This example illustrates the general propensity for defective hybrid materials to contain both a wide diversity of point defects and for correlated defect disorder to emerge from the interactions between defects.

These two trends are just as important for the Prussian Blue analogue (PBA) family of cyanide frameworks. In Prussian Blue itself, the substitution of Fe^2+^ for Fe^3+^ is compensated by the formation of Fe(CN)_6_^4−^ vacancies:2
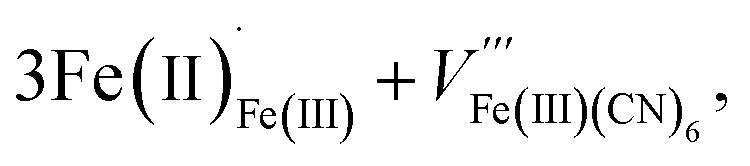
leading to a final composition Fe^III^_4_[Fe^II^(CN)_6_]_3_·14H_2_O.^11^ A second composition, 
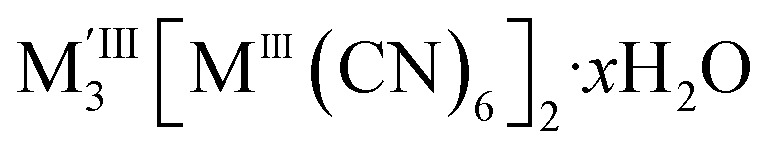
, containing M^III^(CN)_6_^3−^ vacancies also commonly occurs in PBAs.^13^ These hexacyanometallate vacancies produce large voids which are filled with water clusters in the as-synthesised materials.^11,13^ Both the vacancies and the water clusters play a critical role in determining the functional properties of these frameworks, including their mechanical robustness,^14^ their stability as pigments^15^ and whether they show photomagnetism.^16^ In addition, the ordering in space of these defects will likely play a key role in mass transport through these materials for applications including ionic conduction and gas sorption.^7,8,17,18^

PBAs also demonstrate one of the significant challenges of studying defective hybrid frameworks: it can be extremely difficult to determine their structures. Although dozens of compositions of PBAs have been reported,^17^ they are typically produced as microcrystalline powders, which inherently limits the amount of information available through diffraction.^19^ Even for single crystal and total scattering studies where information about the correlation of vacancies through the lattice can be obtained, the high symmetry of the parent structure means the local defect-structure determined through crystallography is superimposed on both the non-defective structure and the symmetry-related transformations of the defect, hindering detailed interpretation.^8,13,20,21^ The presence of disorder also complicates the interpretation of spectroscopic data, as deconvolution of different defect sites can often be challenging unless there are additional helpful features such as moderate paramagnetism.^22^ These problems are by no means unique to PBAs^9^ and thus, despite the utility of accurate representations of defect-structures, we are often forced to resort to simplified models which omit the true complexity of defective molecular frameworks.

There is now growing interest in the potential of thiocyanate analogues of PBAs as functional materials,^23,24^ and we have recently shown that hexathiocyanatobismuthate can be a versatile building block for the formation of Prussian Blue/perovskite-type structures M[Bi(SCN)_6_] (M = Sc^3+^, Cr^3+^, Fe^3+^).^25^ The thiocyanate anion imparts strong optical absorption to the framework through ligand to metal charge transfer bands and also introduces large octahedral tilts through the bent Bi–Ŝ–C bond angle, thereby significantly reducing both the symmetry and volume of the frameworks.

**Fig. 1:**
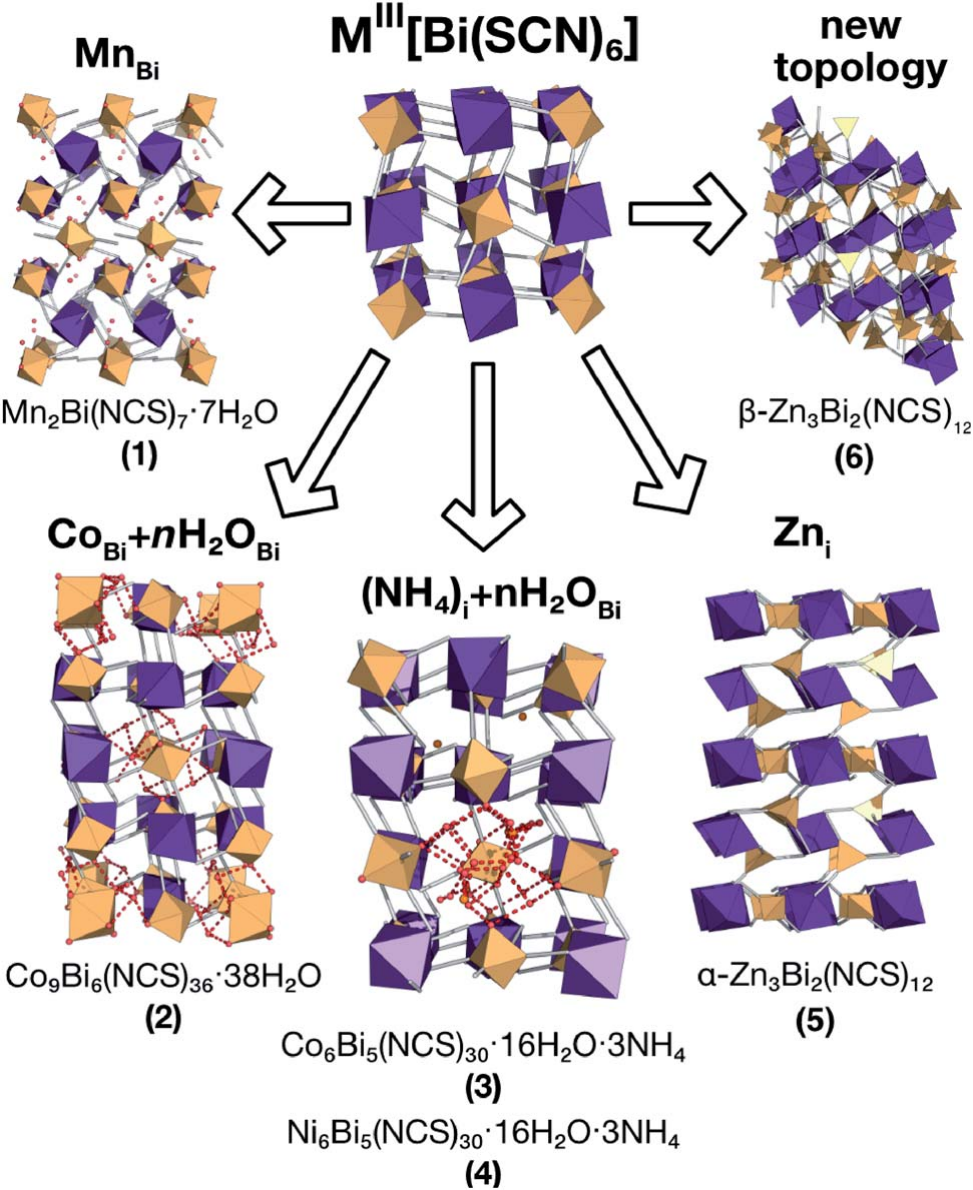
Summary of the new defect-ordered thiocyanate Prussian Blue structures. Colour scheme: purple, Bi; orange, transition metal; NCS ligand, grey; and H_2_O, NH_4_ and hydrogen bonds, red.

In this paper we investigate the defect chemistry of thiocyanate analogues of Prussian Blues by investigating the divalent analogues with the 
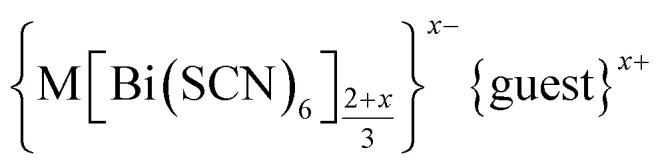
, M = Mn^2+^, Co^2+^, Ni^2+^ and Zn^2+^ [Table 1 and Fig. 1], in part inspired by previous partial reports of other transition metal thiocyanates hexathiocyanatobismuthates.^26,27^ This aliovalent substitution introduces a high concentration of defects which, when combined with the low symmetry of the parent structure, produces long-range defect order. The presence of long-range order allows us to carry out detailed crystallographic investigations of both the identities and distribution of defects. We find that despite the close similarities between the chemistry of these transition metals, the defect structures are diverse, in both local structure and long-range order. We show that the point defects in these frameworks include [Bi(SCN)_6_]^3−^ vacancies, water clusters of up to 20 molecules, anti-site defects, and incorporation of interstitial charge-balancing cations. These point defects order into complex superlattice patterns which are, to the best of our knowledge, unknown in perovskites, both hybrid and conventional, and which suggest new routes to hybrid improper ferroelectrics. We also find that where lower coordination-numbers are feasible for the transition metal, *e.g.* Zn^2+^, new ordered structures can be favoured over defective frameworks, while retaining the strong optical absorption of their non-defective parents. The range of chemistry found in these materials suggests that control over defects could produce surprising new functionality throughout molecular perovskites.

**Table 1:** Summary of reported compounds^a^

	Formula	*x* b	*f* c	Guest(s)
1	Mn_2_Bi(SCN)_7_·7H_2_O	0		Mn(NCS)_2_(OH_2_)_4_·10(H_2_O)^d^

2	Co_9_Bi_6_(SCN)_36_·(H_2_O)_38_	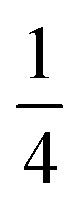	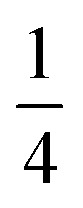	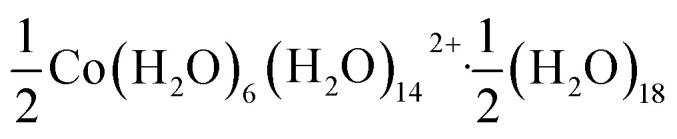

3	Co_5_Bi_6_(SCN)_30_·2NH_4_·16H_2_O·X^e^	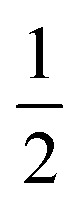	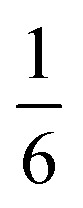	[(H_2_O)_16_X^+^]·2(NH_4_)^+^^e^

4	Ni_5_Bi_6_(SCN)_30_·2NH_4_·16H_2_O·X^e^	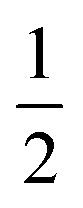	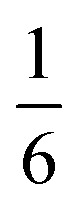	[(H_2_O)_16_X^+^]·2(NH_4_)^+^^e^

5	α-Zn_3_Bi_2_(SCN)_12_	0	—f	—f

6	β-Zn_3_Bi_2_(SCN)_12_	0	—f	—f

a Generic composition 
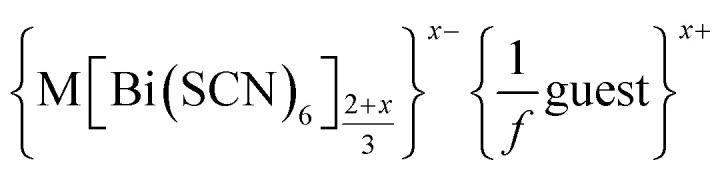
.

b *x* is the charge on the anionic framework charge per (framework) transition metal.

c The vacancy fraction, 
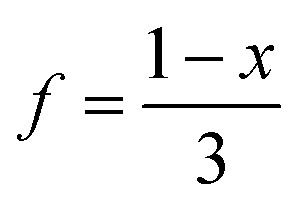
.

d There is disorder over the position of thiocyanate and water, see Section 2.1 for further details.

e Cation X is likely to be NH_4_^+^ but may be H_3_O^+^, see Section 2.3 for further details.

f 5 and 6 do not contain vacancies or guests.

## 2 Results and discussion

### 2.1 Compound 1: Mn_2_Bi(SCN)_7_·7H_2_O

**Fig. 2:**
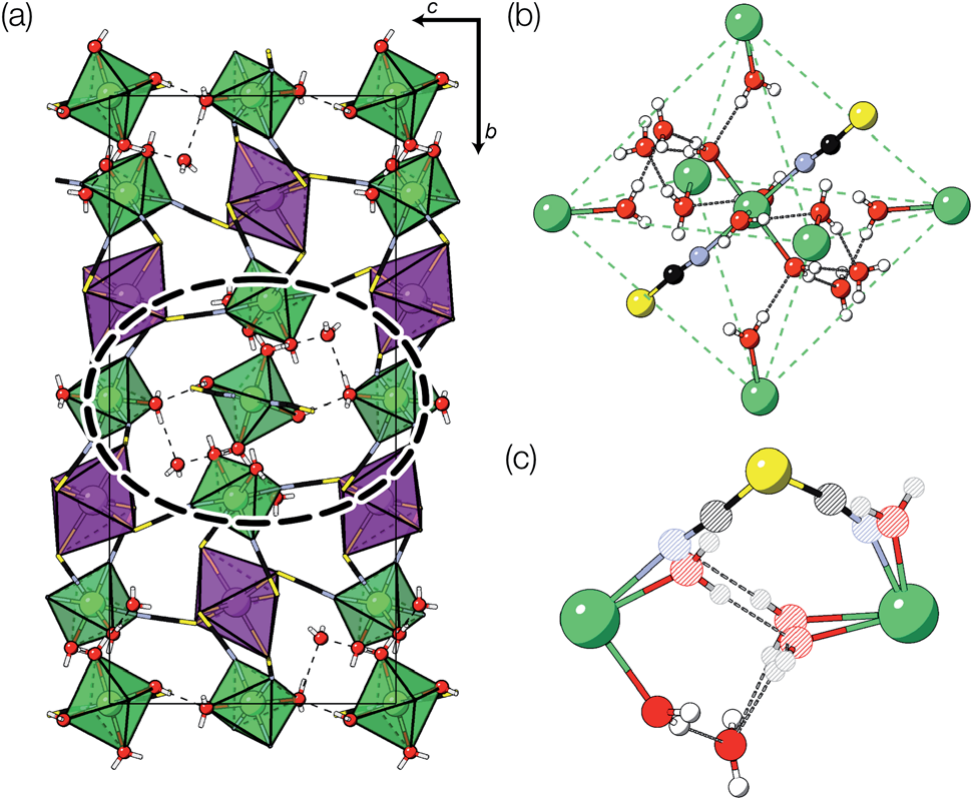
(a) Crystal structure of Mn_2_[Bi(SCN)]_6_(NCS)·7H_2_O (1) at 300 K. A dashed ellipse highlights the manganese molecular complex, shown in greater detail in (b). Disorder around the molecular cluster has been omitted in both (a) and (b) to aid visualisation. (c) A close-up view of the occupational disorder of the NCS and H_2_O ligands in the vicinity of the molecular cluster. Colour scheme: purple, Bi; green, Mn; N, blue; C, black; S, yellow; red, O and white, H. Dashed lines indicate H-bonds, and partially occupied sites are indicated by striped circles.

Evaporation of a saturated solution of manganese carbonate in concentrated aqueous H_3_[Bi(SCN)_6_] yields diffraction-quality single crystals of Mn_2_[Bi(SCN)_6_](NCS)·7H_2_O, 1, (synthetic details are available in the ESI†). Single crystal structure determination at 180 K revealed that 1 is a defective thiocyanate Prussian Blue, where the aliovalent substitution of Mn(ii) for every M(iii) is compensated for by the introduction of one Bi(SCN)_6_^3−^ vacancy on one third of the Bi(iii) sites [Fig. 2(a)]. These Bi(SCN)_6_^3−^ vacancies are occupied by molecular aqua-Mn(ii) thiocyanate complexes with average composition Mn(H_2_O)_5_(NCS)^+^, connected to the framework by hydrogen bonds [Fig. 2(b)]. The positive charge on the molecular complex is balanced by an additional terminal framework thiocyanate which replaces a bound water. The molecular Mn(ii) complex is disordered and the charge-balancing thiocyanate are disordered, with the site bound to the framework occupied by water and thiocyanate in a 0.528(16) : 0.472(16) ratio, and the site bonded to the molecular Mn(ii) complex showing the reverse. When the temperature of the crystal was raised from 180 K to 300 K this ratio was found to be instead 0.70(3) : 0.30(3), indicating that a higher proportion of the NCS ligands are now bound to the framework, which suggests that there may be dynamic effects. The presence of disorder means that it was not possible to determine the relative proportions of the Mn(H_2_O)_5_(NCS)^+^ Mn(H_2_O)_6_^2+^ or Mn(H_2_O)_4_(NCS)_2_ complexes [Fig. 2(c)].

**Fig. 3:**
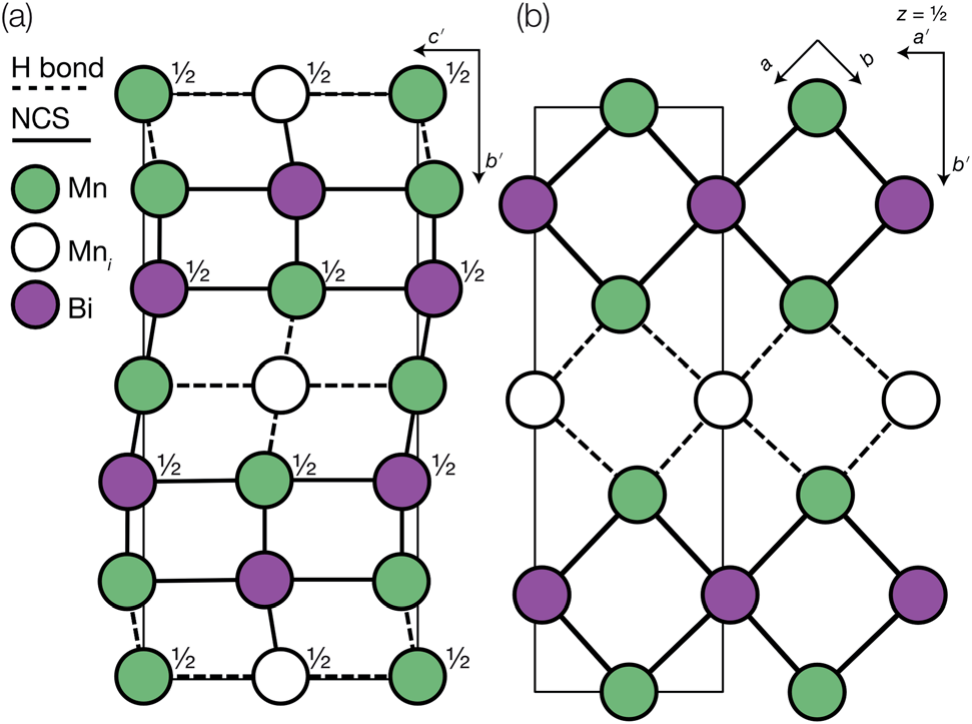
Defect ordering pattern for 1. (a) A view along the *a* axis. (b) A slice through the *ab*-plane, with 
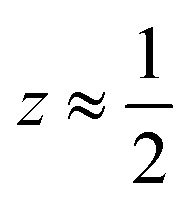
.

The Bi(SCN)_6_^3−^ vacancies are long-range ordered and lie within every third (010) layer of metal atoms, corresponding to a {110}-type layer in the primitive cubic BX_3_ aristotype. Within these (010) planes, all the Bi(SCN)_6_^3−^ anions are missing, so the structure comprises two successive non-defective layers followed by a third defective layer containing only Mn metal ions [Fig. 3]. This defect ordering pattern lowers the symmetry of the cubic aristotype to the orthorhombic *Immm* space group. When combined with the cooperative octahedral tilts produced by the bent M-SCN bonding, the structure lowers to the observed *P*2_1_/*n* space group. We confirmed that the observed framework structure is related to the parent structure within the descent-of-symmetry framework by using the ISODISTORT software package and Topas Academic [ESI Video 1†].^28–30^ The resulting cell can be related to the *Pm*3̄*m* aristotype cell by the following transformation3
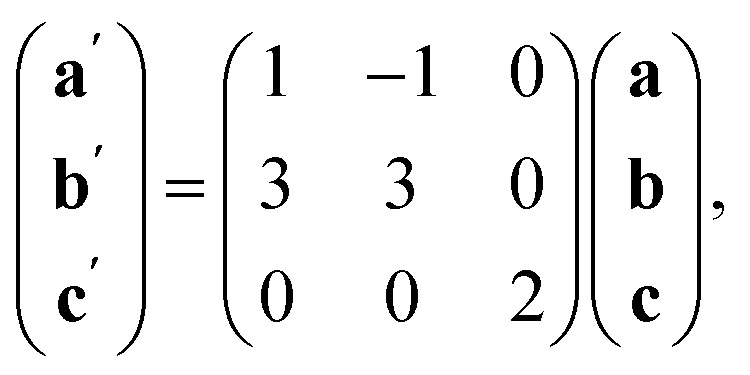
and is a 1 × 3 × 1 supercell of the parent M[Bi(SCN)_6_] structure.

### 2.2 Compound 2: Co_9_Bi_6_(SCN)_36_·(H_2_O)_38_

**Fig. 4:**
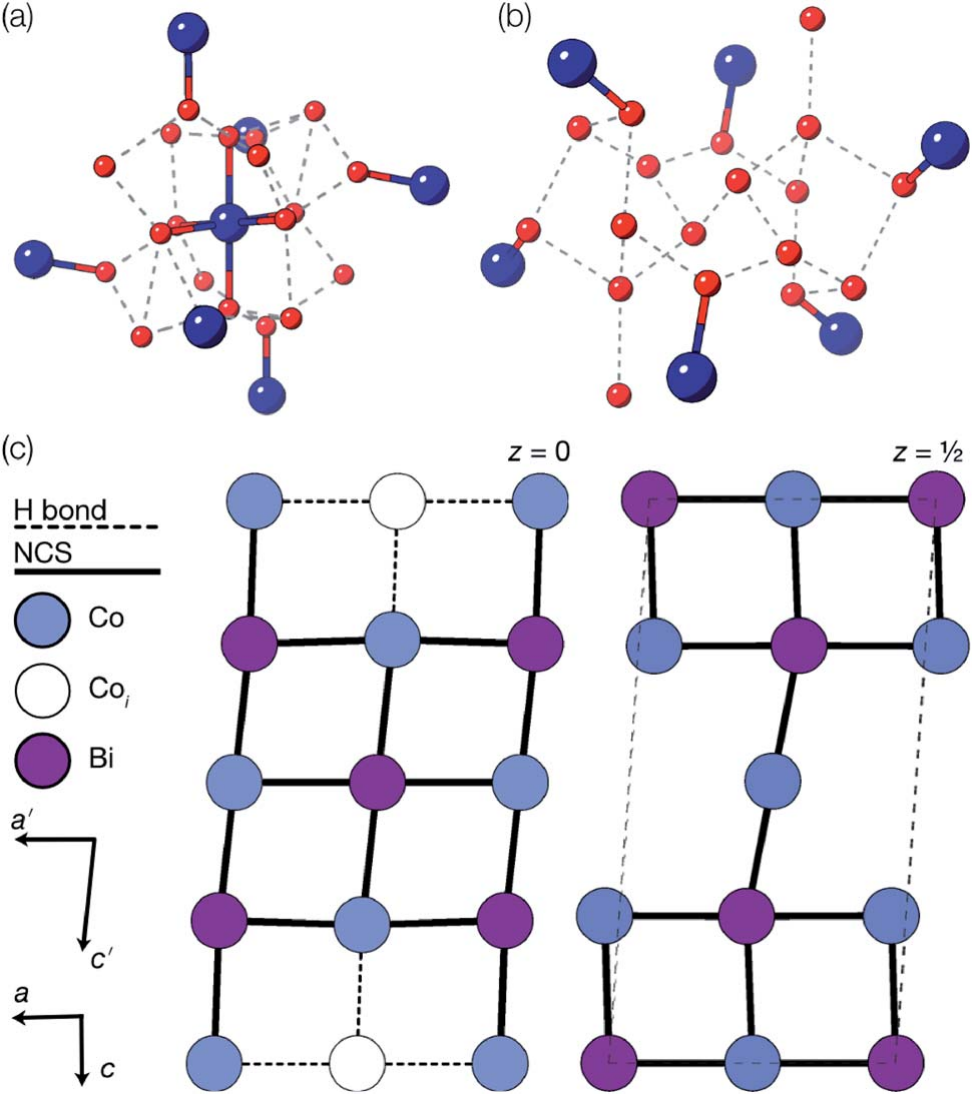
Water clusters present in 2: (a) Co^II^(H_2_O)_6_·14H_2_O and (b) 18H_2_O. Colour scheme: blue, Co and red, O. Dashed lines indicate H-bonds. (c) Ordering pattern for 2, slice with *z* = 0 and 
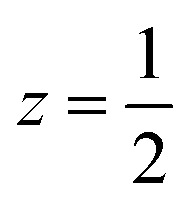
.

Using an analogous route to the synthesis of 1 but substituting basic cobalt carbonate for manganese carbonate, we were able to synthesise a new framework of composition Co_9_Bi_6_(NCS)_36_(H_2_O)_38_ (2). Compound 2 also has a defective Prussian Blue structure with missing hexathiocyanatobismuthate anions, however the mechanism by which this is accommodated is quite distinct from that of 1. In 2 Bi(SCN)_6_ vacancies order into alternate (001) layers, within which half of all Bi(SCN)_6_ polyhedra are absent in a chequerboard fashion. This gives a total vacancy concentration of 
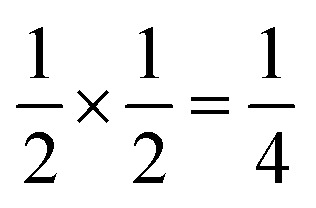
 [Fig. 4(c)]. The vacant sites are then filled by large 18 molecule water clusters in one half of the (001) layers and in the other half by charge balancing hydrated metal cations, Co(H_2_O)_6_^2+^·14H_2_O cations [Fig. 4(a and b)].

Significant disorder is present for both the (H_2_O)_18_ and Co(H_2_O)_6_^2+^·14H_2_O clusters, which prevents the determination of the hydrogen atom positions due to the large atomic displacement parameters. The complexity of the network means that the exact hydrogen bonding pattern remains unknown, though it is clear from the distances between O atoms and their geometric arrangement that significant hydrogen bonding is present. Six waters in each cluster coordinate to the framework Co(ii) atoms, completing the octahedral coordination environment of the metal atoms. Comparison of the bond-valence sums of each of the Co atoms with literature values confirms that all framework transition metal atoms remain in the Co(ii) oxidation state. The hexaaquacobalt complex which occupies half the Bi vacancy sites has very enlarged atomic displacement parameters and significantly lengthened Co–O bond lengths compared to similar complexes. Elemental analysis (energy-dispersive X-ray spectroscopy on crystalline samples and ICP-OES on the framework dissolved in HNO_3_) confirmed the absence of other adventitious cations such as Ca^2+^ and refinement of the structure with hexaquabismuth(iii) produced a significantly worse quality of fit to the experimental diffraction data. As the refined structure shows no other defects and in the absence of other plausible candidates, we therefore believe this cation is likely to be Co(ii).

The observed defect ordering would, on its own, reduce the symmetry of the primitive cubic aristotype to a 2 × 2 × 4 *I*4/*mmm* structure, and when combined with octahedral tilting, this yields the resultant 2 × 2 × 4 *P*1̄ symmetry [ESI Video 2†]. This structure relates to the M[Bi(SCN)_6_] structure through the following transformation matrix:4
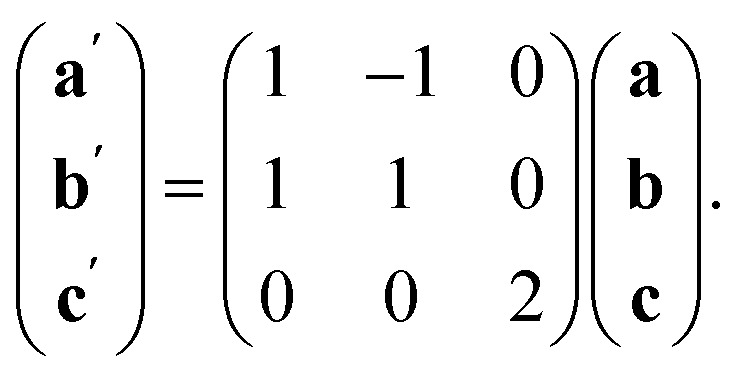


### 2.3 Compounds 3 and 4: M_5_Bi_6_(SCN)_30_·2NH_4_·16H_2_O·X, M = Co & Ni

We also explored the synthesis of frameworks using NH_4_SCN. We found that by using NH_4_SCN as a source of SCN^−^ and reacting it with Co(NO_3_)_2_ and Bi(NO_3_)_3_ using dilute HNO_3_ as a solvent, small, diffraction quality single crystals of a new phase, Co_6_Bi_5_(SCN)_30_·16H_2_O·3NH_4_ (3), formed rapidly, which incorporates NH_4_ cations. Whereas our attempts to synthesise Ni-based frameworks using HSCN and basic Ni salts failed, we were able to use this new route to produce a new framework isostructural to 3, Ni_6_Bi_5_(SCN)_30_·16H_2_O·3NH_4_ (4). Single crystal diffraction revealed that 3 and 4 are defective perovskite frameworks containing Bi(SCN)_6_^3−^ vacancies, the structures of which can be thought of, like 2, as {100} perovskite blocks separated by vacancy containing layers. Just as in 2, these Bi(SCN)_6_^3−^ vacancies order in a chequerboard fashion in (100) layers, however, only every third layer contains vacancies and so the total vacancy concentration is 
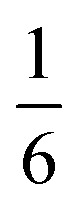
.

**Fig. 5:**
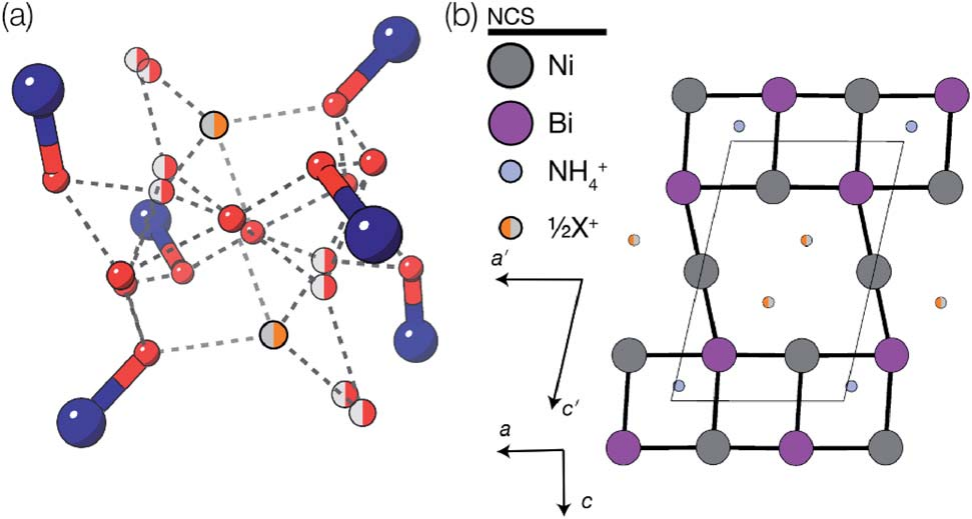
(a) The 16(H_2_O)·X^+^ cluster present in structure 3 (isostructural to that present in 4). Colour scheme: dark blue, Co(Ni); red, O and orange, X. Sites with half occupancy (*e.g.* O, X) are shown as split, dashed lines indicate H-bonds. Hydrogen atoms on the bound waters could not be located in the single crystal electron density maps and are not shown. (b) Ordering pattern for 3 and 4, slice with *z* = 0. Half occupied X^+^ sites are shown as half filled orange circles.

These vacancies compensate for half the negative charge introduced by substituting M(ii) for M(iii). The remaining charge is compensated by interstitial cations, which occupy two different sites. The first site lies between two non-defective layers, and is occupied by ammonium. For compound 4 we were able to locate the H-atoms in the electron density difference map, allowing us to confirm this assignment. The second site forms part of the large 18 atom water cluster (Fig. 5). This water cluster, like that in 2, shows significant disorder, in part because each cluster lies across a centre of symmetry but contains only one cation, ‘X^+^’. The presence of the cation was inferred from charge balance constraints, and we decided which of the electron density peaks was the cation on the basis of two factors: as it is half-occupied it will be the source of the disorder and it will be a better hydrogen bond donor than the water molecules. As we were unable to locate the H-atoms, definitive assignment of X^+^ was not possible, and although it is likely to be NH_4_^+^ as it is present in large excess in the synthesis mixture, the high acidity of the synthesis conditions mean that H_3_O^+^ is also a feasible candidate. This water cluster also coordinates to the Co sites which would be otherwise under-coordinated due to the Bi(SCN)_6_^3−^ vacancies.

The ordering of the Bi vacancies on the primitive lattice lowers the symmetry to *Cmmm* producing a 6 × 2 × 2 supercell compared to *Pm*3̄*m*, which then is lowered by the octahedral tilting, which has Glazer notation *c*^−^*b*^+^*a*^−^ [ESI Video 3†], to a triclinic cell related to the *Pm*3̄*m* by the following transformation matrix:5
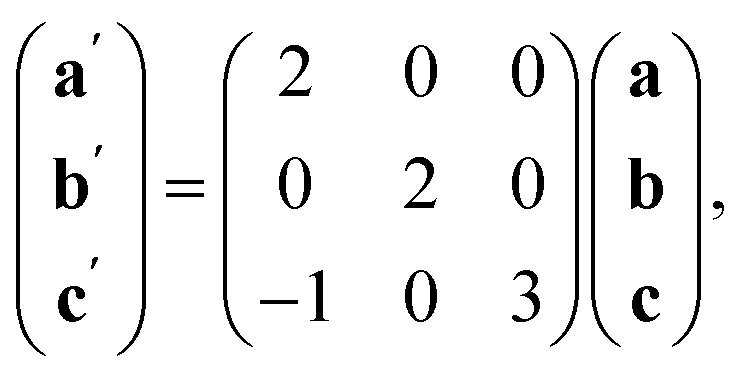
and to the M[Bi(SCN)_6_] structure by6
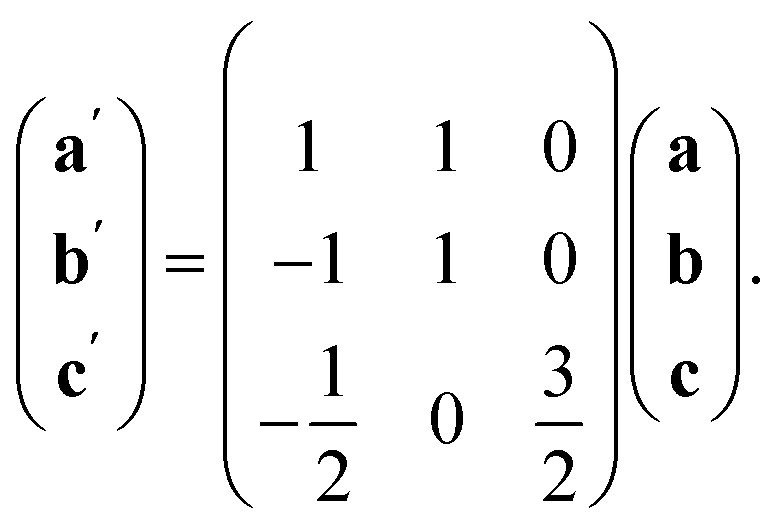


### 2.4 Compounds 5 and 6: Zn_3_[Bi(SCN)_6_]_2_

**Fig. 6:**
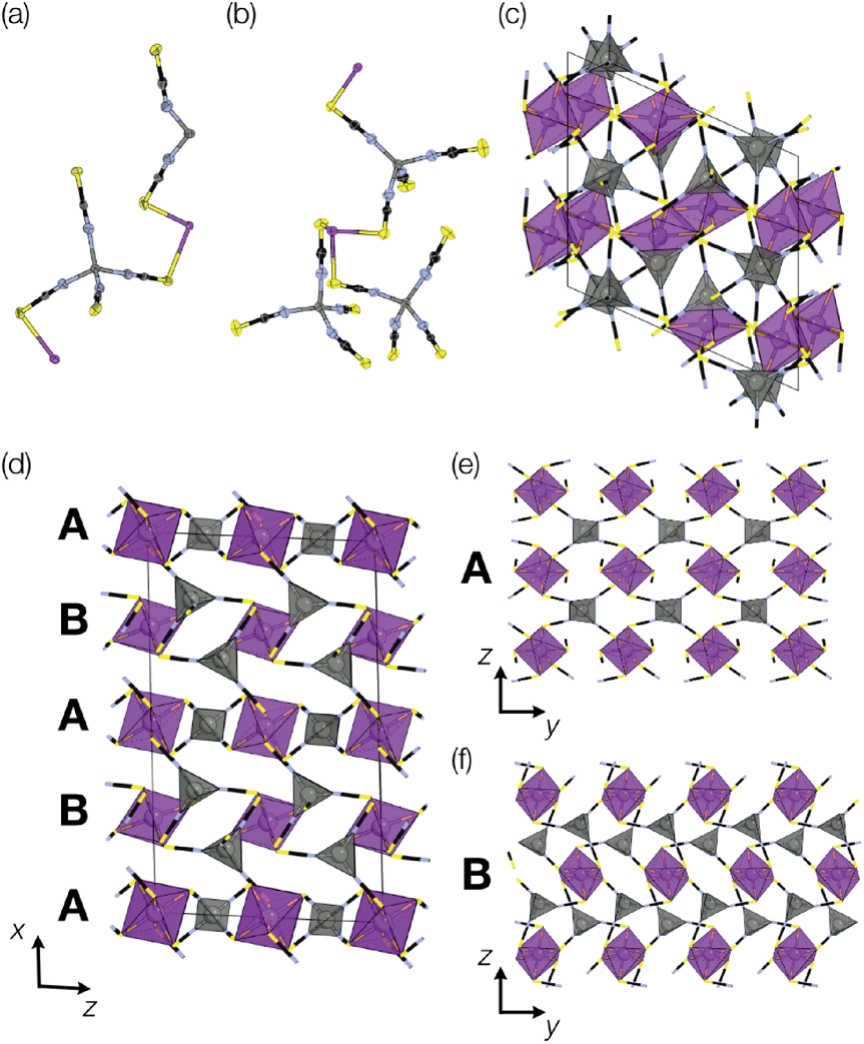
Structures of α- and β Zn_3_Bi_2_(SCN)_6_. Colour scheme: purple, Bi; grey, Zn; yellow, sulfur; blue, nitrogen and black, carbon. (a) Asymmetric unit of α-Zn_3_Bi_2_(SCN)_6_, (b) asymmetric unit of β-Zn_3_Bi_2_(SCN)_6_. (c) Unit cell of β-Zn_3_Bi_2_(SCN)_6_, with coordination polyhedra drawn for the BiS_6_ and ZnN_4_. (d) Unit cell of α-Zn_3_Bi_2_(SCN)_6_, viewed along the *b* direction, highlighting the alternating sequence of *bc*-layers along the *a* direction. The two kinds of layers are shown along *b*-direction showing (e) the pseudo-perovskite A and (f) pseudo-CdI_2_ B.

Addition of Zn(NO_3_)_2_ to a dilute HNO_3_ solution of H_3_[Bi(SCN)_6_] prepared from NH_4_SCN produced single crystals over a period of minutes, and by picking out single crystals we were able to solve two distinct polymorphic frameworks using X-ray diffraction, α-Zn_3_[Bi(SCN)_6_]_2_ (5) and β-Zn_3_[Bi(SCN)_6_]_2_ (6) [Fig. 6]. Despite the similarity in synthesis route to that used for 3 and 4, these new phases do not incorporate NH_4_^+^ from solution. In addition, unlike the other phases we describe, 5 and 6 do not contain lattice water and their structures are much less closely related to that of Fe[Bi(SCN)_6_]. Rietveld refinement of a polycrystalline sample confirmed that the polymorphs crystallise concomitantly, with the predominant fraction consisting of the β phase (92%) [ESI Fig. 1†]. Careful examination of the powder X-ray diffraction data showed that the product contains at least one additional phase which we have been unable to isolate. As with the Prussian Blue derived frameworks, the bent NCS–Bi bond angle leads to the formation of low symmetry structures, with multiple symmetry distinct metal sites in each (in α, two Bi and two Zn sites, and in β two Bi and three Zn sites).

The local connectivity is identical between the two phases, and there are only minor differences in bond lengths and angles. Despite these similarities, the topologies of framework connectivity of the α and β phases are very different. Indeed, by using the ToposPro software, we were able to determine that the graphs produced by considering the Zn_3_Bi_2_ sublattice for both α- and β-Zn_3_[Bi(SCN)_6_]_2_ possess topologies which were not yet recorded in the ToposPro topology database.^31^ We have termed these new topologies **clw1**, point symbol: {4^3^.6^3^}2{4^4^.6^1^0.8}{4^4^.6^2^}{4^6^.6^6^.8^3^} and **clw2**, point symbol: {4^2^.6^4^}{4^3^.6^3^}{4^4^.6^11^}{4^4^.6^2^}{4^5^.6^9^.8}.

The α phase is derived from the parent phase by the incorporation of Zn^2+^ interstitials in half of all layers. More completely, it is built from two different layers, a Prussian Blue-like layer and CdI_2_-like layer, which stack in an alternating fashion along the *a* axis to form a three-dimensional framework [Fig. 6(d)]. The Prussian Blue-like layer has the composition ZnBi(SCN)_2_ and can be described as a {100} layer of a hypothetical ‘ZnBi(NCS)_6_’ analogue of the Fe[Bi(NCS)_6_] structure [Fig. 6(e)]. As the Zn is four-coordinated, its bonding requirements are saturated within the layer, and so the layer is connected to the layers above and below through the Bi atom. The second layer has composition Zn_2_Bi(NCS)_6_·NCS and a pseudo-CdI_2_ structure, where the Bi (‘Cd’) atoms approximately form a triangular lattice, in which the interstices are occupied by Zn (‘I’) atoms [Fig. 6(f)]. In this layer, the Bi atoms are saturated by bonds within the layer, and so the connection to the other layer occurs through the Zn atoms, which are bound to an additional thiocyanate.

The predominant size of ring in the β-Zn_3_Bi_2_(NCS)_12_ phase is the sixteen-membered Zn_2_Bi_2_(NCS)_4_ ring (four-membered ring, considering only metal cations) common to the M[Bi(SCN)_6_] perovskite. The ring-statistics are otherwise very different [Fig. 6(c)] and the structure cannot be easily decomposed into simple crystal-chemical units. The coordination geometry for both α and β phases remain similar, and there is only slightly larger variation in the octahedral coordination of BiS_6_ distortions in the β phase.

The differences observed between Zn-based frameworks and the other transition metals derives from the increased favourability of the tetrahedral coordination, which permits the formation of charge-neutral and coordinatively saturated frameworks from Zn(NCS)_4_ tetrahedra and Bi(SCN)_6_ octahedra without the need for point vacancies or coordinating water molecules. This is directly akin to the cyanide Prussian Blue family, for which Zn_3_[Fe(CN)_6_]_2_ can form both an ordered phase in which zinc is tetrahedrally coordinated, and a defective disordered cubic phase, analogous to the other Prussian Blues, in which Zn is octahedrally coordinated.^32^ In Zn_3_[Fe(CN)_6_]_2_, the polymorphism occurs between a disordered and an ordered phase, whereas both polymorphs of Zn_3_[Bi(SCN)_6_]_2_ are ordered.

### 2.5 Optical properties

Each of these new phases possesses a very strong coloration, as found in the parent M[Bi(SCN)_6_] family.^25^ We carried out initial diffuse reflectance spectroscopy measurements which allowed us to quantify the optical absorption onset. They revealed that in each case the band gap is close to that observed for Sc[Bi(SCN)_3_],^25^ suggesting that the transition is primarily LMCT in character: from the HOMO of NCS^−^ to the empty Bi p-states [Table 2]. However the band gaps of 1 and 2 are slightly smaller than those of the other compounds and weak sub-band gap absorptions are seen, suggesting that metal d-states may mix with the Bi p-states and that d–d transitions also contribute to the observed spectra [ESI Fig. 3 and 4†].

**Table 2:** Experimental optical band gaps^a^

	1	2	4	5 & 6^b^
*E*_g_ (eV)	2.13(5)	2.24(5)	2.30(5)	2.28(5)

a Estimated standard errors in parentheses. Optical band gaps determined using a direct band gap Tauc plot [ESI Fig. 3].

b Sample consisted of a mixture of compounds 5 & 6.

## 3 Discussion

To the best of our knowledge, the only previous reports of any compounds belonging to this family were by Cygańksi,^26,27^ but in these early studies the analysis was confined principally to determining their composition. The following compositions were reported: MnBi(SCN)_5_, Mn_3_[Bi(SCN)_6_]_2_·7H_2_O, Mn_3_[Bi(SCN)_6_]_2_·12H_2_O, Ni_3_[Bi(SCN)_6_]_2_·10H_2_O, which do not correspond to any of the phases uncovered in our study; Co_3_[Bi(SCN)_6_]_2_·12H_2_O which corresponds approximately to 2, and Zn_3_[Bi(SCN)_6_] which corresponds to 5 and 6. The fact that these early studies report compositions we have not yet synthesised ourselves indicates that perhaps there is even more latent complexity in this family of materials than we have uncovered.

Aside from 6, all structures retain strong similarities to the parent Fe[Bi(SCN)_6_] structure, despite the stark differences in composition. If the unit cell volume is normalised by the number of Bi atoms, this normalised volume is within a few percent of Fe[Bi(SCN)_6_] for every compound, with the biggest discrepancy (5.5%) found for compound 6. This suggests that the high vacancy concentration does not produce significantly contracted frameworks, which is likely due to the fact that potential voids are filled by water clusters and molecular complexes. For the compounds containing octahedrally coordinated transition metal ions, 1–4, the pattern of tilts observed in the parent structure is retained [ESI Videos 1–3†]. In addition, the (large) magnitude of the octahedral tilting is not significantly perturbed, as the primary source of tilts is the shape of the frontier orbitals of NCS^−^ and the resultant bent Bi–S–C bond angle. The retention of tilt-cooperativity in each of 1–4 is more surprising and is probably due in part to the presence of three dimensional connectivity of octahedra combined with the fact that no framework node has lower than four-fold coordination. These factors suggest that this Fe[Bi(SCN)_6_] structure type is robust and likely to be found in other members of this family and that it may be possible to synthesise porous analogues, without large guests or water clusters.

**Fig. 7:**
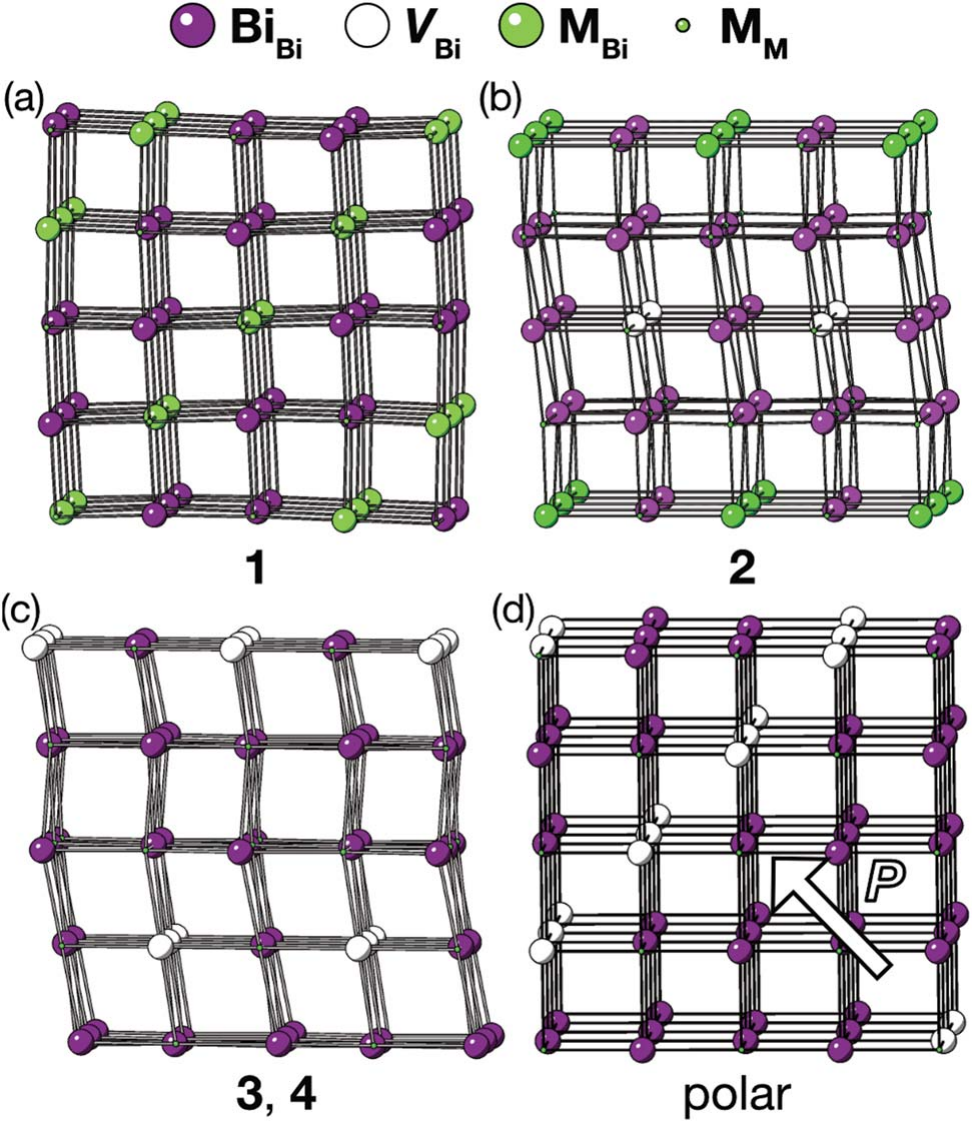
Diagrams illustrating vacancy ordering patterns for compounds (a) 1 (b) 2 (c) 3 & 4 and (d) for a hypothetical four-fold doubling vacancy-ordering which generates a net polarisation when combined with the *a*^−^*a*^−^*c*^+^ octahedral tilts. The polar direction is illustrated by an arrow. Only metal cations are shown in this representation, and bonds are drawn between nearest-neighbour metals whether or not they are connected by a thiocyanate ligand.

The vacancy orderings found in compounds 1–4 are relatively complex for perovskite-type materials [Fig. 7] and lead to, in combination with the retained cooperative octahedral tilts, large and low-symmetry unit cells. Although cation ordering and octahedral tilting are unable to generate polarisation on their own, the ability of certain combinations of A- and B-site cation order and octahedral tilting to generate polar structures is well established, *e.g.* the combination of layered ordering of the A-site cations, rock-salt ordering of B-site cations and *a*^−^*a*^−^*c*^+^ tilts generate a polar structure in space group *P*2_1_.^33^ The use of simultaneous cation order and octahedral tilts to generate ‘hybrid improper’ ferroelectrics has been shown to be particularly efficacious for layered perovskite-derived materials.^34–36^ Although compounds 1–4 all crystallise in non-polar space-groups, the unusual vacancy orderings we observe suggest that a new route to hybrid improper ferroelectricity: combining octahedral tilts with complex vacancy order. Indeed, we predict that a vacancy ordering pattern closely related to that of compound 1, consisting of a four layer repeat of {110} type vacancy containing layers, will produce polar order in combination with the retained *a*^−^*a*^−^*c*^+^ found in each of these phases [Fig. 7(d)], despite neither the vacancy-ordering or tilting producing polarisation on their own. Future synthetic efforts should therefore be alive to the possibilities of new vacancy orders.

The introduction of a divalent metal such as Mn^2+^ onto a trivalent metal site, M, introduces a single net negative charge, in the Kröger–Vink notation, 
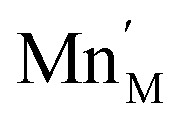
. As the framework is an overall charge-neutral insulator, the framework must compensate by introducing charge balancing defects, and we found that there are a wide variety of mechanisms. α-Zn_3_[Bi(SCN)_6_] can be thought of as containing Zn^2+^ interstitials:7
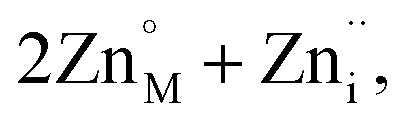


For the other transition metals, the transition metals retain octahedral coordination, even though tetrahedral Co(ii) is well-known, and so they adopt structures more closely related to the M[Bi(SCN)_6_] structure. Compounds 1–4 all contain Bi(NCS)_6_^3−^ vacancies, and a key difference in these structures is how the resultant voids are filled and the open structures are stabilised. This is also a feature of the cyanide PBAs, which contain significant quantities of zeolitic water.^37^ In these thiocyanate frameworks the guest species are considerably more varied, and include molecular metal complexes with water and thiocyanate as ligands. These species may be more challenging to realise in PBAs, as cyanide tends to be a more strongly binding ligand than thiocyanate, and thus water will likely be less competitive as a co-ligand. However, the high symmetry of PBAs hinders detailed study of guest species, and so it is feasible that molecular metal complex guests are present in some PBAs, but have evaded definitive identification. The defect chemistries of compounds 1–4 can be described as follows. Compound 1 contains molecular Mn(NCS)_2_(H_2_O)_4_ complexes in the *V*_Bi(SCN)_6__ sites which are hydrogen-bonded to the network, instead of water clusters:8
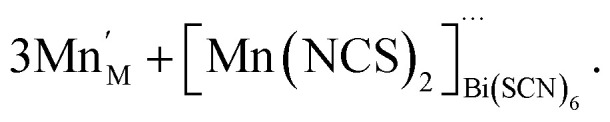


Compound 2, like 1, contains anti-site transition metal cations, which occupy half the *V*_Bi(SCN)_6__ sites. However, these Co^2+^ cations are not coordinated to thiocyanates, and water clusters occupy the remaining half of *V*_Bi(SCN)_6__ sites:9
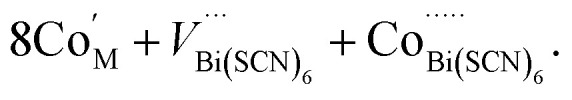


The presence of other interstitial cations, such as NH_4_^+^ in 3 and 4, adds an additional source of complexity, just as found in the cyanide Prussian Blues. Both 3 and 4 compensate for the effective negative charge of the divalent cation by the formation of *V*_Bi(SCN)_6__, which are all filled with water clusters, and ammonium cations:10
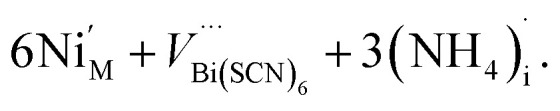


The ability of the thiocyanate perovskite framework to accommodate monovalent A-site cations has been previously demonstrated for CsCd(NCS)_3_ (ref. 38) and (NH_4_)_2_CdNi(NCS)_6_,^24^ however the large molecular guests found in 1 and 2 imply that it may be possible to deliberately include large molecular species in framework cavities. Including functional molecular complexes as guests would be a powerful route for introducing useful properties. Polar molecular complexes or organic cations could be a source of electrical polarisation and light-emitting or photocatalytic metal complexes could make use of the strong absorption of the metal-thiocyanate framework. In particular, we anticipate further investigations into the optical properties of these materials will shed light on the ways that both framework topology and guest–framework interactions can be used to control the electronic structure and optical properties of this family of materials.

The diversity of guests and vacancy orderings we have found in this family of chemically similar materials suggests that the factors dictating which phase forms are relatively subtle. The notable differences in the aqueous chemistry of divalent transition metal thiocyanates, *e.g.* the ready hydration of Co(NCS)_2_ compared to Ni(NCS)_2_,^39,40^ suggest that the study of solution-phase thiocyanate complexes may provide valuable insights in the origins of variation in the solid state and suggest future routes to generate novel structures.

## 4 Conclusion

We have found six new framework compounds of the approximate formula 
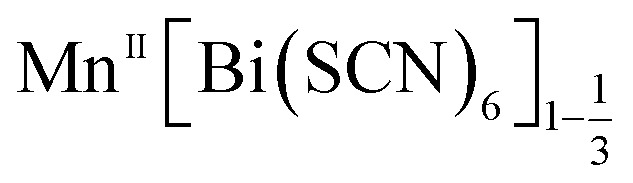
. The primary mechanism by which the charge of aliovalent M(ii) cations is accommodated by the frameworks is through the introduction of Bi(SCN)_6_ vacancies, analogous to the M(CN)_6_ vacancies found in the Prussian Blue; however anti-site defects, large water clusters and even neutral and cationic molecular metal complexes are also accommodated. In addition to this, we find that the low symmetry of the structure enables long-range order of these defects: vacancies tend to segregate into {100} or {110} type layers. The complexity of these structures is such that single-crystal X-ray diffraction data was required to uncover it.

Despite the relatively high concentration of vacancies, we also found that the octahedral tilt patterns of the pattern structures are retained in all the Prussian Blue-derived members of this family, showing their robust nature. Compounds 3 and 4 demonstrate that as in the Prussian Blues, the presence of other cations can have a powerful effect on the resultant structure, as their incorporation leads to drastically different ordering patterns. The observed combination of complex vacancy order and retained cooperative octahedral tilts may produce new routes to hybrid improper ferroelectricity.

In conclusion, we have shed light on the surprising complexity of aliovalent doping in this family of hybrid framework materials. We anticipate that as the investigation of hybrid frameworks advances, the range of defects we have uncovered in thiocyanate frameworks will be reflected in other anion chemistries. These novel defect types will have different energetic formation costs, and the complex orderings will perturb the cooperative properties, such as mechanical flexibility, magnetism or electrical polarisation. The adaptability of these structures further suggests that guests of significant complexity such as molecular complexes could be included in these frameworks to introduce new functional behaviour, whether directly in synthesis or post-synthetically through ion-exchange processes.

## Conflicts of interest

There are no conflicts of interest to declare.

## Supplementary Material

SC-011-D0SC01246G-s001

SC-011-D0SC01246G-s002

SC-011-D0SC01246G-s003

SC-011-D0SC01246G-s004

SC-011-D0SC01246G-s005
