# Supplementary material for: The structures of ordered defects in thiocyanate analogues of Prussian Blue
Source: Chem Sci. 2020 Apr 9;11(17):4430–8. doi: 10.1039/d0sc01246g (PMC8159453; doi:10.1039/d0sc01246g)
Supplement: SC-011-D0SC01246G-s001 [file SC-011-D0SC01246G-s001.pdf]

Electronic Supplementary Material (ESI) for Chemical Science.
This journal is © The Royal Society of Chemistry 2020

The structures of ordered defects in thiocyanate Prussian Blue analogues

Matthew J. Cliffe,[*a,b] Evan N. Keyzer,[a] Andrew D. Bond,[a] Maxwell A. Astle[b] and Clare P. Grey[a]

April 6, 2020

[a] Department of Chemistry, University of Cambridge, Lensfield Road, Cambridge CB2 1EW, UK;
[b] School of Chemistry, University of Nottingham, University Park, Nottingham NG7 2RD, UK;
*To whom correspondence should be addressed; e-mail: matthew.cliffe@nottingham.ac.uk.

Contents

1 Synthetic Procedures **2**
1.1 Synthesis of HSCN 2
1.2 Synthesis of $H_3[Bi(SCN)_6]$ solution 2
1.3 Synthesis of **1** 2
1.4 Synthesis of **2** 2
1.5 Synthesis of **3** and **4** 2
1.6 Synthesis of **5** and **6** 2

2 Single Crystal X-ray Diffraction **3**

3 Powder X-ray Diffraction **5**

4 ICP-OES **6**

5 STEM-EDX **6**

6 Diffuse Reflectance **8**

List of Figures

1 Rietveld refinement of mixed phase α- and β $Zn_3Bi_2(SCN)_6$. * indicates the presence of an impurity peak. 5
2 Representative EDX spectrum for compound **2** 7
3 Tauc normalised diffuse reflectance data for determination of band gap **1**,**2**,**4**,**5** & **6** 8
4 Diffuse reflectance spectra for compounds **1**,**2**,**4**,**5** & **6** 9

List of Tables

1 Summary of key crystallographic parameters for all compounds 4
2 STEM-EDX elemental ratios in sample **2** 6

1 Synthetic Procedures

The synthesis procedures were adapted from those reported in[S1].

1.1 Synthesis of HSCN

In a 250 mL round bottom flask, NH_4SCN (5g, 65.7 mmol) was dissolved in 5 mL H_2O and cooled to 0 °C in an ice bath. A H_2SO_4 solution (ca. 7 ml of H_2SO_4 in 12 ml H_2O) was then added dropwise to the cooled NH_4SCN solution. The reaction mixture was stirred for 30 mins before being warmed to room temperature. The aqueous mixture was subsequently extracted with diethyl ether (2×20 ml) and the organic phase was retrieved and its volume reduced by half using a stream of N_2.

1.2 Synthesis of $H_3[Bi(SCN)_6]$ solution

$Bi_2O_2(CO_3)$ (0.50 g, 0.98 mmol) was suspended in ca. 12 mL H_2O followed by the addition of the HSCN/ether solution. The resulting reaction mixture was stirred vigorously under a slight flow of N_2 until all ether had been removed and the solution had turned bright orange. Any remaining solids were filtered off and the orange solution was placed under a slight vacuum to remove any excess HSCN.

1.3 Synthesis of 1

1mL of the prepared $H_3[Bi(SCN)_6]$ was added to approximately 50 mg $MnCO_3$, and left to react overnight. Any excess solids were removed by gravity filtration. The dark red solution was then left to evaporate in a watch glass covered by petri-dish for a period of approximate two weeks until diffraction quality dark orange single crystals formed.

1.4 Synthesis of 2

1mL of the prepared $H_3[Bi(SCN)_6]$ was added to approximately 50 mg ($Co_5(CO_3)_2(OH)_6$) and left to react overnight. Any excess solids were removed by gravity filtration. The dark red solution was then left to evaporate in a watch glass covered by petri-dish for a period of approximate two weeks until diffraction quality dark orange single crystals formed.

1.5 Synthesis of 3 and 4

$Bi(NO_3)_3 \cdot 2.5\,H_2O$ (3 mmol, 1.46 g) was dissolved in 1.5 mL 3M HNO_3, and a solution of NH_4SCN (13.1 mmol, 1.00 g) dissolved in 2 mL of distilled water was added to it, producing a vivid orange solution. $Ni(NO_3)_2 \cdot 6\,H_2O$ (7 mmol, 2.036 g) was dissolved in 2 mL of water and then added to to bismuth thiocyanate solution, which on standing produced numerous small very dark orange single crystals of **4** over a period of 15 min. The same route can be used to produce **3**, substituting $Co(NO_3) \cdot 6\,H_2O$.

1.6 Synthesis of 5 and 6

$Bi(NO_3)_3 \cdot 2.5\,H_2O$ (3 mmol, 1.46 g) was dissolved in 1.5 mL 3M HNO_3, and a solution of NH_4SCN (13.1 mmol, 1.00 g) dissolved in 2 mL of distilled water was added to it, producing a vivid orange solution. $Zn(NO_3)_2 \cdot 6\,H_2O$ (7 mmol, 2.082 g) was dissolved in 2 mL of water and then added to to bismuth thiocyanate solution, which on standing produced an immediate precipitate of numerous orange single crystals of **5** and **6**.

2 Single Crystal X-ray Diffraction

Single crystals were selected and mounted using perfluorinated oil on a polymer-tipped micromount and cooled rapidly to measurement temperature 120 K or 180 K in a stream of cold N_2 using an Oxford Cryosystems open flow cryostat. To enable variable temperature measurements, the crystal used for structures **1** and **1a** was mounted using varnish on a pin.

Single-crystal X-ray diffraction data for **2**, **4**, **5** and **6** were collected using a Nonius KappaCCD diffractometer, using graphite monochromated MoKα radiation (λ = 0.7107 Å). Data for **1** and **1a** were collected using a on a Bruker D8-Quest PHOTON-100 diffractometer equipped with an Incoatec IμS Cu microsource ($\lambda = 1.54056$Å). Data for **3** were collected using Single crystal X-ray diffraction data were collected on an Oxford Diffraction GV1000 (AtlasS2 CCD area detector, mirror-monochromated Cu-K$_\alpha$ radiation source ($\lambda = 1.54184$Å). Structure solution was carried out using SHELXT and refinement with SHELXL, within the OLEX2 graphical interface.[S2–4] For crystal **1**, hydrogen atoms were refined with constrained geometries and riding thermal parameters, however the disorder present in samples **2**–**4** meant that hydrogen atoms could not be located, aside from an NH_4 cation in **4**. Disordered sites in structures **1**, **2**, **3** and **4** were modelled at half occupancy.

ESI Table 1: Summary of key crystallographic parameters for all compounds

Compound	**1**	**1a**	**2**
Formula	$Mn_2Bi(SCN)_7\,7\,H_2O$	$Mn_2Bi(SCN)_7\,7\,H_2O$	$Co_9Bi_6(SCN)_{36}\,(H_2O)_{38}$
Molar mass	851.54	851.54	4535.47
Crystal System	Monoclinic	Monoclinic	Triclinic
Space Group	$P2_1/n$	$P2_1/n$	$P\bar{1}$
Crystal Colour	dark orange	dark orange	dark orange
Z	4	4	1
Radiation	Cu K_α	Cu K_α	Mo K_α
Temperature (K)	300	180	180
a (Å)	8.3698(3)	8.3065(2)	12.0209 (2)
b (Å)	26.0037(9)	25.8428(6)	12.1613 (2)
c (Å)	12.2466(4)	12.2664(3)	23.8319 (4)
α (Å)	90	90	94.200 (1)
β (Å)	91.208(2)	90.2360(10)	94.003 (1)
γ (Å)	90	90	91.452 (1)
V (Å^3)	2664.83(16)	2633.12(11)	3464.52 (10)
Measured reflections	30850	33300	21789
Independent reflections	4712	4657	13585
R_{int}	0.1315	0.080	0.035
$R[F^2 > 2\sigma(F^2)]$	0.0553	0.038	0.059
S	1.013	1.03	1.13
CCDC number			

Compound	**3**	**4**	**5**	**6**
Formula	$Co_5Bi_6(SCN)_{30}\,3\,NH_4 \cdot 16\,H_2O$	$Ni_5Bi_6(SCN)_{30}\,3\,NH_4 \cdot 16\,H_2O$	α-$Zn_3Bi_2(SCN)_{12}$	β-$Zn_3Bi_2(SCN)_{12}$
Molar mass	3438.91	3512.13	1311.03	1311.03
Crystal System	Triclinic	Triclinic	Monoclinic	Monoclinic
Space Group	$P\bar{1}$	$P\bar{1}$	$C2/c$	$P2_1/c$
Crystal Colour	dark orange	dark orange	orange	orange
Z	1	1	4	4
Radiation	Cu K_α	Mo K_α	Mo K_α	Mo K_α
Temperature (K)	120	111	109	180
a (Å)	11.9106 (3)	11.8567 (2)	26.3104 (4)	17.7331 (6)
b (Å)	11.9382 (3)	11.8653 (2)	8.4587 (1)	13.6501 (4)
c (Å)	8.4250 (5)	18.3824 (4)	15.7403 (3)	16.4375 (4)
α (Å)	83.935 (2)	84.0731 (7)	90	90
β (Å)	76.504 (2)	76.7140 (7)	93.630	114.3971 (12)
γ (Å)	85.384 (2)	85.1942 (8)	90	90
V (Å^3)	2529.04 (12)	2498.60 (8)	3496.00 (9)	3623.55 (19)
Measured reflections	37154	20614	7507	12934
Independent reflections	9921	11356	3975	7822
R_{int}	0.047	0.050	0.029	0.052
$R[F^2 > 2\sigma(F^2)]$	0.031	0.043	0.026	0.051
S	1.03	1.04	1.07	1.05
CCDC number				

Cu K_α, $\lambda = 1.54056$Å, Mo K_α, $\lambda = 0.71073$Å.

3 Powder X-ray Diffraction

A high-resolution synchrotron X-ray powder diffraction measurement on a ground powder of $Zn_3Bi_2(NCS)_{12}$ was carried out at beamline 11-BM at the Advanced Photon Source (APS) using a wavelength of 0.414537 Å. The sample was loaded into a 0.8 mm diameter Kapton capillary. Rietveld refinement of the data was carried out using Topas Academic 4.1.[S5, S6] Lattice parameters were allowed to refine freely along with isotropic displacement parameters for Bi atoms and terms accounting for crystallite size broadening and crystallographic strain. The presence of a minor tertiary phase was modelled using independently refining peaks, which we were unable to index as a separate phase.

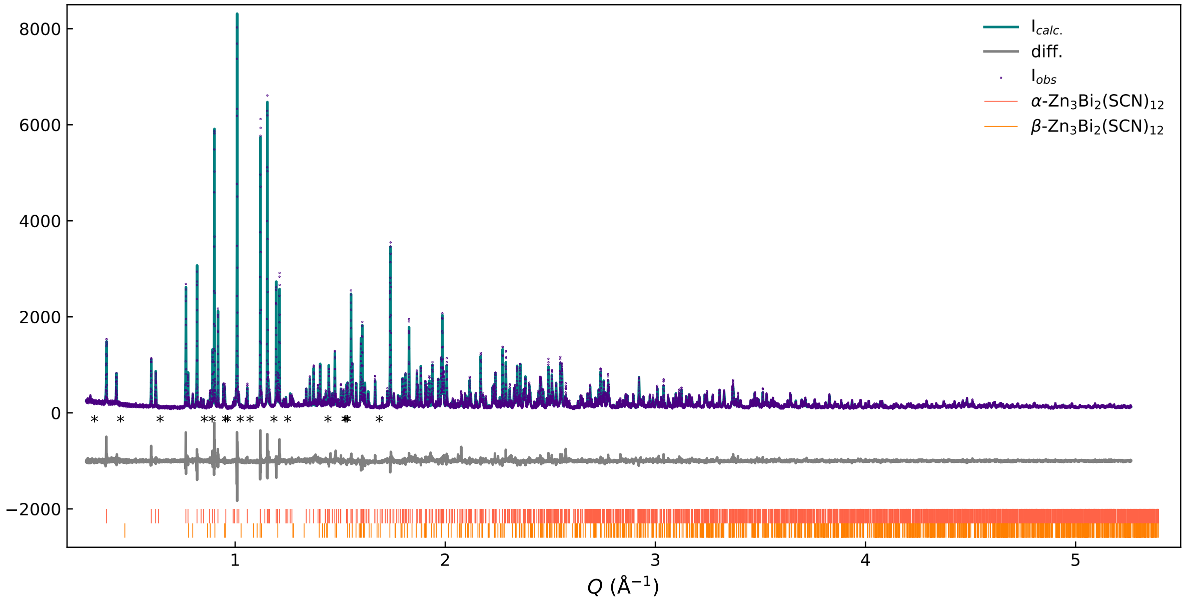

ESI Fig. 1: Rietveld refinement of mixed phase α- and β $Zn_3Bi_2(SCN)_6$. * indicates the presence of an impurity peak.

4 ICP-OES

Inductively coupled plasma optical emission spectroscopy (ICP-OES) was recorded on a Perkin Elmer, Optima 2000 DV ICP-OES with S10 autosampler. Samples and standards were prepared with a final solution composition of 2% nitric acid. Metal content was kept below 50 mg/L for ICP measurements as at higher concentrations a precipitate would form which would not dissolve in limited volumes of acids. Found for sample **2** Bi 27.35%, Co 17.97%, Ca 0.0%, Ni 0.0% and Ti 0.0%.

5 STEM-EDX

Energy dispersive X-ray (EDX) spectroscopy was acquired using dark field scanning transmission electron microscopy (STEM), performed using a JEOL JEM-2100+ microscope operated at 200 kV and an Oxford Instruments XMaxN 100TLE X-ray microanalysis system. Samples were deposited onto copper grid mounted "lacey" carbon films (Agar) and the beam was condensed to areas suspended over holes of the amorphous carbon to negate the contribution to the carbon signal from the support film. Copper contributions from the grid were discounted from the analysis. The measured proportions of light atoms (C, N and O) will have contributions from the carbon film and as these measurements were carried out under high vacuum, it is likely a significant proportion of lattice water will have been lost.

ESI Table 2: STEM-EDX elemental ratios in sample **2**

Element	Wt%	At%	At. Ratio	At. Ratio (calc.)
Bi	19.87	2.02	2	2
Co	9.74	3.51	3.47	3
S	20.14	13.32	13.19	12
C	23.9	42.18	41.76	12
N	21.61	32.71	33.39	12
O	4.72	6.25	6.19	12.67
Ca	0.02	0.01	0.01	0
Ti	0	0	0	0
Ni	0	0	0	0

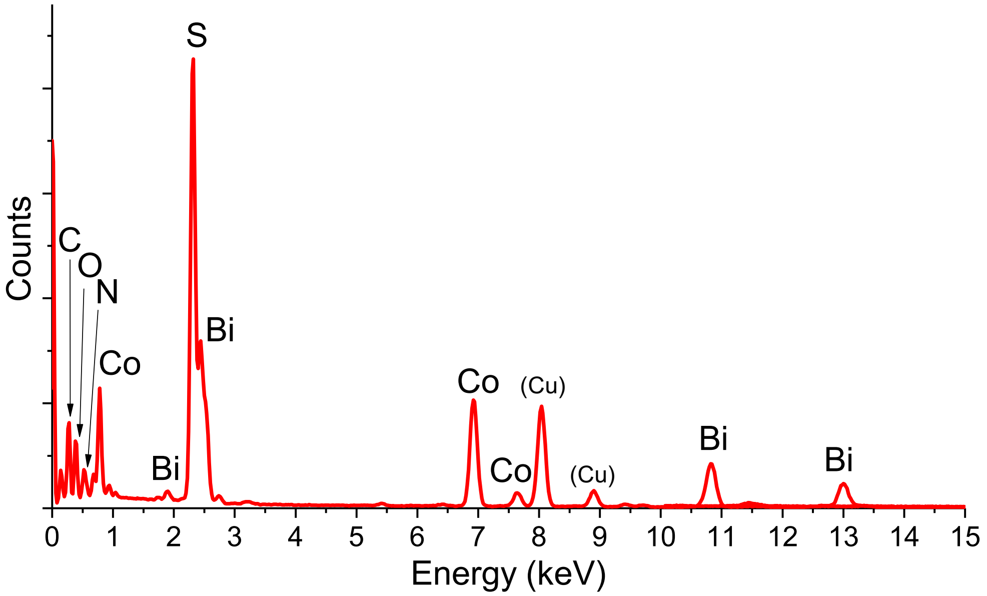

ESI Fig. 2: Representative EDX spectrum for compound **2**

6 Diffuse Reflectance

Diffuse reflectance measurements were carried out on finely ground powdered samples, diluted to 10 wt% with $BaSO_4$ to remove effects of strong absorption, using a Varian Cary 50 UV-vis spectrometer equipped with a diffuse reflectance accessory (DRA) probe (Barrelino, Harrick Scientific) measured over the range 350–1000 nm. All measurements were performed under standard ambient conditions. The absorption onsets were determined by extrapolation from a Tauc plot, using the Tauc exponent for a direct band gap.

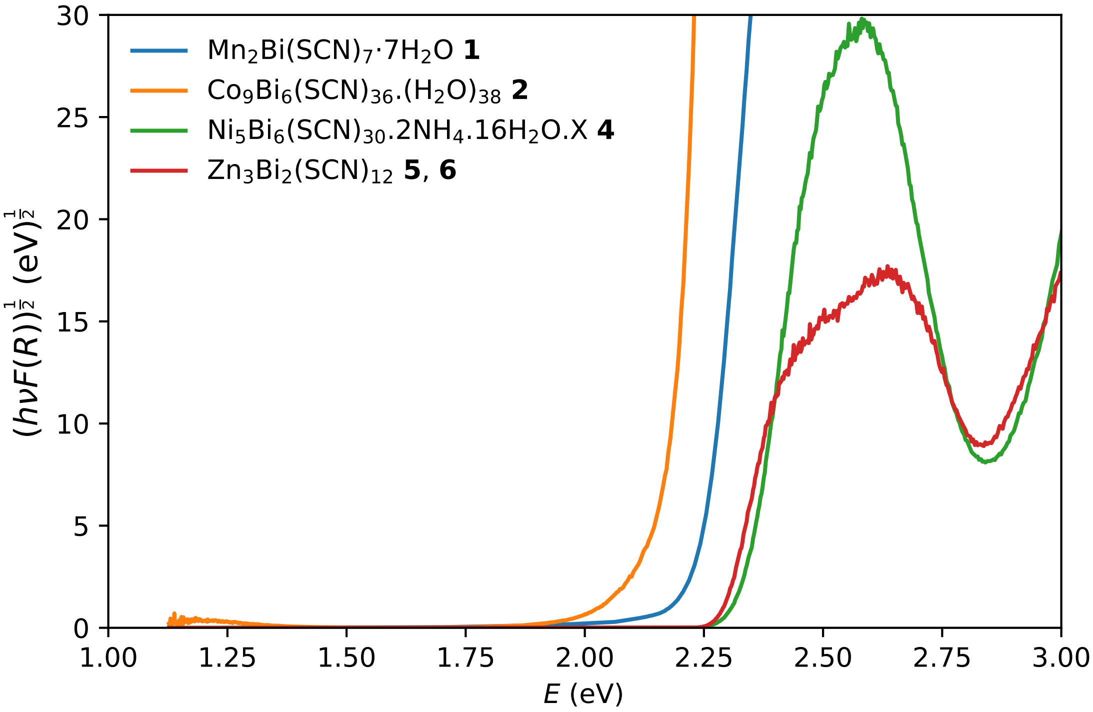

ESI Fig. 3: Tauc normalised diffuse reflectance data for determination of band gap **1**,**2**,**4**,**5** & **6**

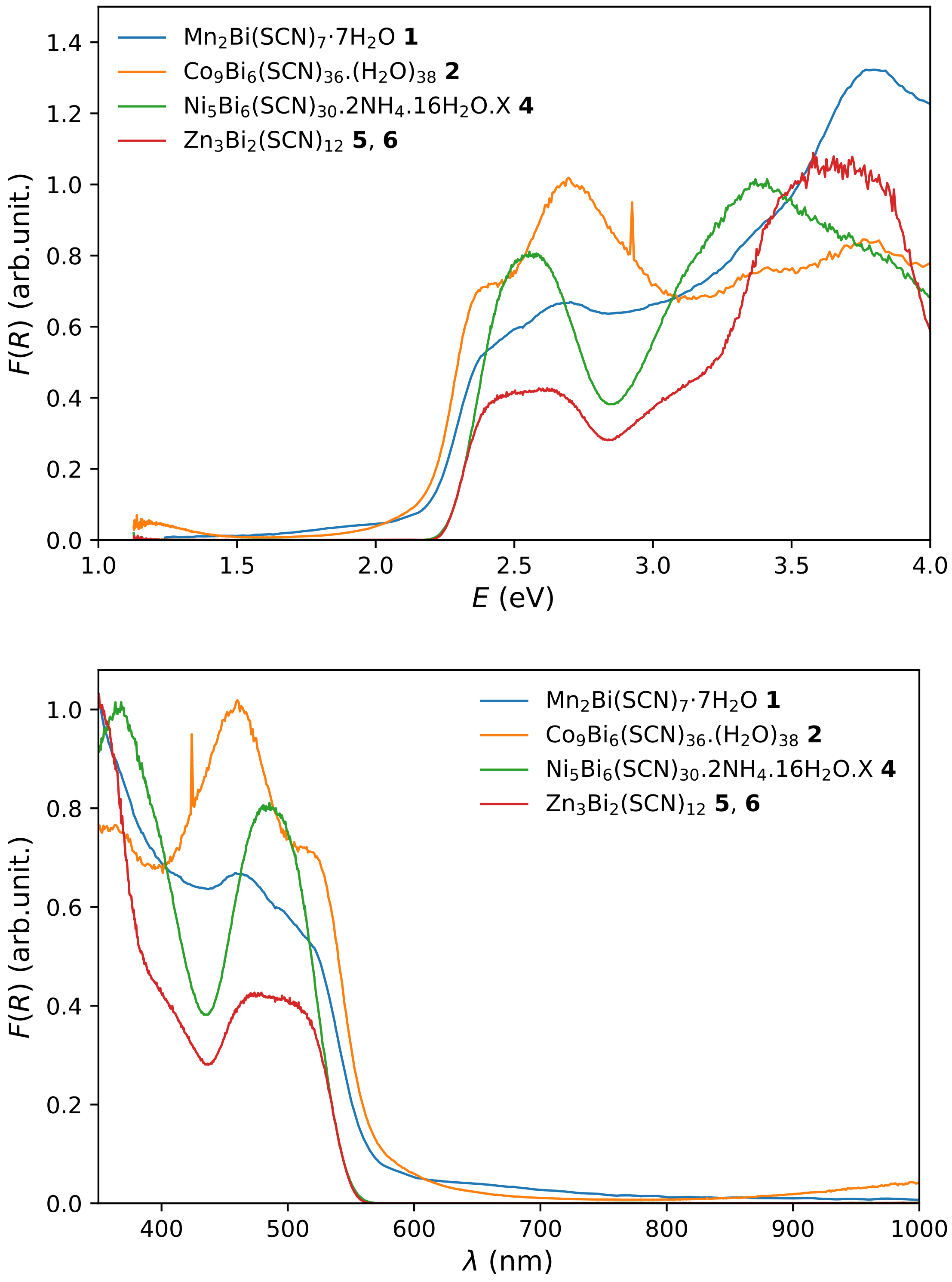

ESI Fig. 4: Diffuse reflectance spectra for compounds **1**,**2**,**4**,**5** & **6**

References

(S1) M. J. Cliffe, *et al.*, *Chemical Science* **10**, 793 (2019).
(S2) O. V. Dolomanov, L. J. Bourhis, R. J. Gildea, J. A. K. Howard, H. Puschmann, *J. Appl. Crystallogr.* **42**, 339 (2009).
(S3) G. M. Sheldrick, *Acta Crystallogr.* **A71**, 3 (2015).
(S4) G. M. Sheldrick, *Acta Crystallogr.* **C71**, 3 (2015).
(S5) H. Rietveld, *J. Appl. Crystallogr.* **2**, 65 (1969).
(S6) A. Coelho, *J. Appl. Crystallogr.* **36**, 86 (2003).
